# Did We or Didn't We? Louse Genetic Analysis Says Yes

**DOI:** 10.1371/journal.pbio.0020378

**Published:** 2004-10-05

If you're an evolutionary biologist, the tired old saw, “You can tell a lot about a person by the company they keep,” represents a fresh new approach to a longstanding problem. Especially if the company in question is a parasite—say, for example, lice—and the problem is tracing the path of human evolution.

One of the hottest debates in the study of human origins centers around whether modern Homo sapiens interbred with archaic humans. While genetic data have provided insight into recent human evolution, fossils remain the only available evidence for many archaic human species—and the fossil record is notoriously spotty, leaving the data open to multiple interpretations. Two prominent models and a subset of variants have emerged, differing mainly on the question of gene flow: one asserts that modern humans emerged from an archaic ancestor in Africa about 130,000 years ago and then replaced archaic forms in Africa, Asia, and Europe with no gene flow between them; the other proposes gene flow between modern human populations as well as interbreeding between modern and archaic forms in different parts of the world. Both models find support in the available data, but neither can claim a perfect fit with all the data, leaving the possibility of interbreeding an open question.

Faced with a relative paucity of human fossil and genetic data, scientists have been forced to rely on other data sources. Mounting evidence suggests that parasites with an established coevolutionary history with their hosts can serve as a proxy for host evolutionary history, an especially handy tool in the event of insufficient host data. Following this approach in a new study, David Reed and colleagues circumvent the lack of human data by analyzing the next best thing: head lice.

As host-specific, obligate parasites—that is, occurring on a single species and not able to survive off that host—lice require direct physical contact between hosts for transmission. As human parasites, lice harbor in their genetic sequence hints of the slings and arrows of evolutionary fortune (and touches of grace, for that matter) that strike their host. Recent studies of the evolutionary history of other human parasites (tapeworms, malarial parasites, and human papillomaviruses), for example, fall in line with fossil and genetic data that place our origins in Africa.

**Figure:**
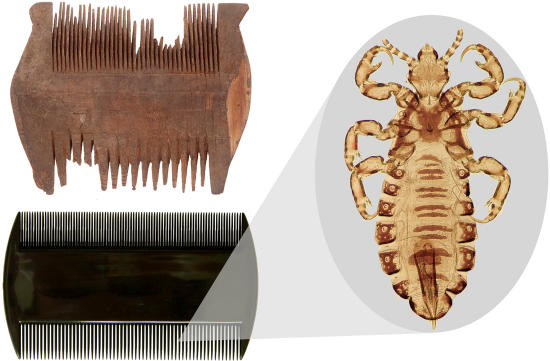
Ancient nit combs (above) resemble modern ones (below). (Egyptian wooden comb courtesy of Te Papa, Wellington, New Zealand, negative number F.003884/5. Modern louse comb and head louse images by Vincent S. Smith)

Two louse species parasitize humans, head/body (Pediculus humanus) and pubic (Pthirus pubis). Head and body lice obviously occupy different habitats, but are not genetically distinct. Interestingly, P. humanus contains two ancient lineages, offering the opportunity to shed light on this murky period in human evolution. To do this, Reed and colleagues had to reconstruct the evolutionary history of P. humanus, which they did using morphological and genetic analyses of this and other species of lice. They confirmed that P. humanus comprises two lineages—one contains both head and body forms and has worldwide distribution; the other contains only the head louse and is restricted to the New World—but discovered that P. humanus originated long before its H. sapiens host.

Humans went through a population bottleneck around 100,000 years ago, followed by an expansion; one would expect to see the same thing in lice. Population genetics studies revealed, however, that only the worldwide lineage went through a bottleneck and subsequent expansion. The New World lineage not only maintained a relatively stable population size but followed an evolutionary path distinct from the worldwide lineage for the past 1.18 million years. It is unlikely, the authors argue, that two ancient louse lineages could embark on such different evolutionary histories on the back (or head) of a single host. More likely, the New World louse evolved on an archaic form of humans and then cast its lot with a modern version.

While the split between H. sapiens and H. neanderthalensis was too recent (about 700,000 years ago) to support a concurrent split between the worldwide and New World lice lineages, the split between H. sapiens and H. erectus (about 1.8 million years ago) could. Reed and colleagues propose a scenario in which H. sapiens and H. erectus carried distinct types of lice owing to a million years or so of isolation. As the first waves of modern humans left Africa about 100,000 years ago and modern humans replaced archaic forms, the two forms engaged in enough contact—whether in the form of fighting, swapping clothes, or interbreeding—for archaic lice to make the switch to modern human hosts. Tackling the question of interbreeding, the authors suggest, might best be pursued by studying P. pubis, which requires sexual contact for transmission.

